# Southwest Greenland shelf glaciation during MIS 4 more extensive than during the Last Glacial Maximum

**DOI:** 10.1038/s41598-019-51983-3

**Published:** 2019-10-30

**Authors:** Marit-Solveig Seidenkrantz, Antoon Kuijpers, Jesper Olsen, Christof Pearce, Sofia Lindblom, Johan Ploug, Piotr Przybyło, Ian Snowball

**Affiliations:** 1 Department for Geoscience, Aarhus University, Høegh-Guldbergs Gade 2, 8000 Aarhus C, Denmark; 2 Geological Survey of Denmark and Greenland (GEUS), Øster Voldgade 10, 1350 Copenhagen K, Denmark; 3 Aarhus AMS Centre, Department of Physics and Astronomy, Aarhus University, Ny Munkegade 120, 8000 Aarhus C, Denmark; 4 Department of Earth Sciences, University of Gothenburg, Sweden, Denmark; 5 DJ Miljø & Geoteknik, Falkevej 12, 3400 Hillerød, Denmark; 6 25 Coleherne Road, Earls Court, SW10 9BS London, United Kingdom; 7 Department of Earth Science, Uppsala University, Villavägen 16, 75236 Uppsala, Sweden

**Keywords:** Palaeoclimate, Palaeoceanography

## Abstract

Although geological and modelling evidence indicate that the last glacial inception in North America was in NE Canada, little is known about the glacial response of the nearby western Greenland Ice Sheet (GIS) during the glacial advance of marine oxygen isotope stage 4 (MIS4). Our multi-proxy study of a marine sediment core collected about 60 km southwest of the Outer Hellefisk Moraines demonstrates that in the southern Davis Strait region the most extreme Greenland shelf glaciation of the last glacial cycle occurred during MIS 4, with another prominent glacial advance at 37–33 kyr BP. During those periods the GIS likely reached the Outer Hellefisk Moraines in this area. Except for these two periods, our data suggest significant advection of relatively warm Irminger Sea Water by the West Greenland Current since MIS 4. This advection likely limited the extent of the MIS2 glaciation on the SW Greenland shelf. Decreased precipitation over southwestern Greenland predicted by atmospheric models as a downstream effect of a much larger MIS2 Laurentide Ice Sheet may have played an additional role.

## Introduction

At the end of the last interglacial (Marine Isotope Stage (MIS) 5e) approximately 115,000 years ago (115 kyr BP, before present), the astronomically-driven decline of northern summer insolation and resulting atmospheric and ocean feedback processes led towards the first step in glacial ice sheet formation in the North American region^1^. This interglacial-to-glacial transition at the end of MIS 5e featured prominent changes in oceanographic circulation in the subpolar North Atlantic^2^, which were characterized by progressive expansion of cold water masses and associated southward advances of the Arctic Front as well as ice sheet expansion during marine isotope stages (MIS) 5b and 4. A rapid expansion of the ice sheet in the early glacial is also indicated by numerical ice sheet model simulations^3^. Moreover, model-based simulations further suggest that the glacial expansion in the Baffin Bay – Labrador Sea region most likely started in the mountain range of Baffin Island^4^. Ice sheet extent culminated during the Last Glacial Maximum (LGM, 23–19 kyr), i.e. during MIS 2.

Many studies have outlined the extent of Quaternary glaciations worldwide and in particular the LGM^5^. Yet, few details are known about the extension of the MIS 4 (71–57 kyr) ice sheets. It has, however, been reported that during MIS 4, an extensive outer shelf glaciation occurred on the western Siberian shelf^6,7^, while northern Siberian mountain glaciation was also large^8^. Both for MIS 4 and the preceding cold MIS5b substage (86.2 kyr model age) the size and location of the early glacial ice sheets could be reconstructed by a combination of geological information and numerical modelling^3^. The latter results indicate that the entire north-eastern North American continental edge was glaciated during MIS4, but that it was not until later, i.e. under MIS2, that the ice sheet expanded into the continental interior. Within the present context it is worth noting that an apparent major collapse of the GIS at the MIS 4 termination presumably led to massive calving of North Greenland glaciers and probably contributed to widespread IRD deposition in the Arctic Ocean at the beginning of MIS 3^7^.

Little information on the previous glacial history is available for West Greenland due to subsequent erosion of pre-existing glacial features. Bedrock exposure data indicate ice-free conditions in coastal regions of Sisimiut, Uummannaq and Upernavik for about 55% of the last 1 million years^9^, suggesting strong variability in the ice sheet extent. Due to a lack of accurate dating, most of the glacial features on the West Greenland shelf have been assumed to have an age corresponding to the LGM. A shelf edge extension of the GIS during the LGM has been suggested by a number of studies^10–13^, although a circum-Greenland GIS shelf-edge extension is still a matter of debate^14^. Moreover, there is notably little information available about the response of the GIS margin during the early stage of the last (Weichselian) glaciation, i.e. during MIS4^14,15^, although the GIS extent during MIS 4 is generally believed to have been smaller than during MIS 2. Most the coastal regions of Greenland were deglaciated during MIS 5e, and glacial expansion onto the shelf may not have commenced until MIS 4^9^.

The West Greenland margin is today strongly influenced by the oceanic conditions. The advection of warm, Atlantic-sourced water transported by the West Greenland Current (WGC) is believed to play an important role as one of the controlling factors for the expansion and retreat of marine-terminating glaciers^16,17^. It is thus very likely that the overall changes in Atlantic Meridional Overturning Circulation (AMOC), and not the least its resulting changes in the warm-water flux to West Greenland, may have played a role in deciding extend of expansion of the GIS onto the shelf.

**Figure 1:**
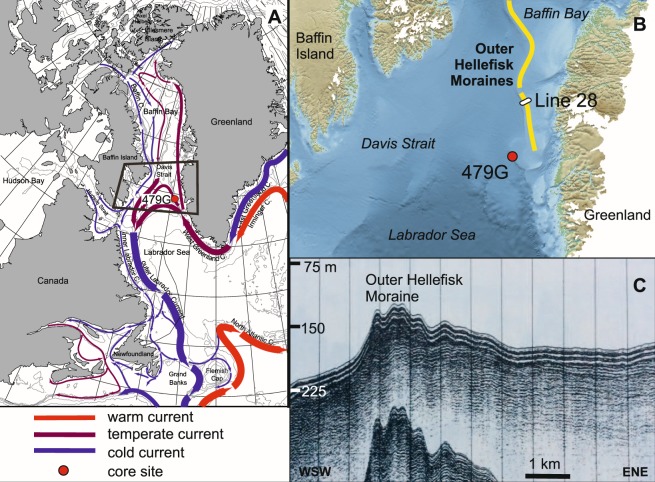
Regional setting of sediment gravity core TTR13-AT-479G (labelled 479 G) located at 64°24.37′N 54°45.08′W, with modern surface ocean circulation pattern (after^68^) and (**B**) indication of the Outer Hellefisk Moraine complex on the Southwest Greenland shelf (based on^18^) as well as (**C**) a sparker record (line 28) illustrating a cross-section of this moraine system at 65° 42.8′N 55° 28.5′W^18^. Location of the sparker section is given in B. Water depth on the site of the moraine is 150–200 m (based on sound velocity 1500 m/s), and moraine height is about 25 m. The moraine deposits are presumably partly covered by a thin veneer of postglacial sediment.

In this study we aim to test for potential periods of extensive shelf glaciations of the GIS during the last glacial cycle, with particular focus on MIS 4 and MIS 2, more in particular for investigating whether the GIS was indeed more extensive during MIS 2 than during MIS 4, as generally assumed. We will further focus on the possible link between shelf glaciation events and changes in the ocean current regime in the north-eastern Labrador Sea (Fig. 1), testing the hypothesis of a strong inverse relationship between Atlantic-sourced water influx and glacial expansion. Our investigations are based on a multi-proxy study based on sedimentological, geochemical, foraminiferal and stable isotope analyses from a marine sediment core, where sedimentological (in particular IRD) and elemental composition from XRF core scanning as well as magnetic properties will inform on the influx of sediments from terrestrial sources, whereas benthic foraminiferal assemblage data supported by planktonic stable isotope data are invaluable for identifying the environmental conditions and changes in ocean circulation.

### Study area and material

A roughly NNW-SSE striking moraine ridge complex is found on the outer West Greenland shelf, previously termed the Outer Hellefisk Moraine system^18,19^ (Fig. 1). Both a Saalian^19^ (MIS6) and a LGM (MIS2) origin^14,20^ have provisionally been proposed. In 2003, the 503 cm long sediment gravity core TTR13-AT-479G (hereafter 479 G) was retrieved from a water depth of 1033 m in the southern Davis Strait near the southwest Greenland shelf edge (64°24.37′N; 54°45.08′W), about 60 km southwest of the Outer Hellefisk Moraine (Fig. 1). The core top seemed intact with limited loss of sediment.

Oceanographically, our study site is today located within the lower depth stratum of the West Greenland Current (WGC) realm (Fig. 1A), a boundary current transporting Atlantic (sub)surface water masses from the Irminger Sea (Irminger Sea Water, ISW) into the northern Labrador Sea, Davis Strait, and eastern Baffin Bay^21^. These relatively warm and saline subsurface waters are overlain by a comparatively thin (<ca. 150 m) layer of cold, low-salinity Polar Water derived from the East Greenland Current and melt water discharge from local South Greenland outlet glaciers. Hence, bottom-water conditions at the site are today influenced primarily by the relatively warm WGC, while surface-water conditions are cooler.

## Results

### Sediments and ice-rafted debris events

**Figure 2:**
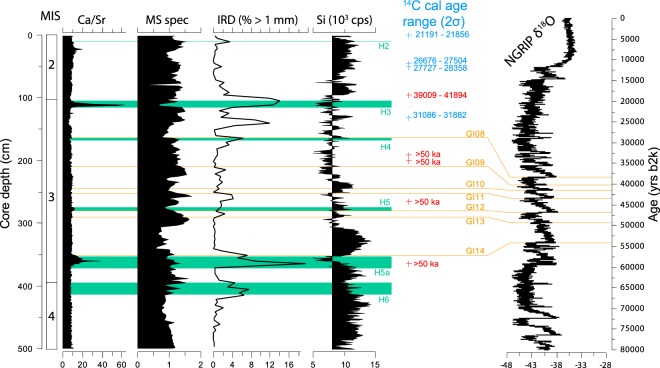
Selected geochemical (Ca/Sr and Si), magnetic (χ, specific magnetic susceptibility) and sedimentological (IRD, > 1 mm) parameters from core TTR13-AT-479G. The correlation to the NGRIP ice core stratigraphy, Greenland Interstadial (GI) chronology (onsets of interstatials, in orange) and Heinrich (H, green intervals) events as well as calibrated 14 C dates (blue: dates included in the age model; red: excluded reversals and infinite ages) are shown. The correlation to Marine Isotope Stages (MIS) is given to the left.

The sediment in gravity core 479 G consists of olive-green to olive-grey silty and sandy clay interrupted by layers of sand and gravel (>1 mm); some levels show a light pink colour in discrete bands (Supplementary material; Fig. S1). Two peaks in coarse grain content, i.e. at ca. 113 cm and 360 cm core depth, coincide with strong maxima in the XRF Ca/Sr record (Fig. 2). Maximum values of Si and K prevail below 300 cm core depth. Marked features in the record of K, Fe and Ti are the occurrence of low concentrations at about 113 cm and 360 cm depth, due to well-defined peaks in the Ca counts (Fig. S2).

**Figure 3:**
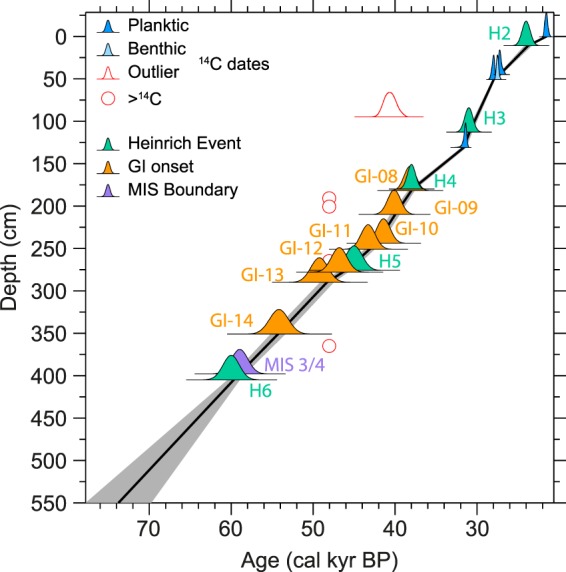
Age model of core TTR13-AT-479G. The age model is based on a combination of ^14^C datings, correlations of IRD peaks, elemental composition to Greenland Interstadials and Heinrich events.

More generally, Ca and Si may be associated with biogenic material from marine primary production (calcareous and siliceous plankton). However, Ca may also originate from detrital carbonate, which is an important component of IRD in the study region^22^, as is reflected in our Ca/Sr record (Fig. 2) which is a well-known proxy of non-biogenic carbonate^23^. Apart from a potential biogenic origin, Si is an important indicator of continent-derived material, and a terrestrial origin of the silica in core 479 G is strongly supported by its similarity to the K record. Such a terrestrial source also applies to K, Fe and Ti^23,24^ (Fig. S2), and off Greenland the Si, K, Fe and Ti records are considered to be linked to glacier melting^25^. In particular, Si and K are indicators for material derived from continental, siliceous rocks, which also makes these important for our age model (see below, Fig. 3). They appear to be overall most abundant in the lower part of core 479 G below approximately 310 cm core depth, but with quite significant fluctuations throughout the length of the core (Fig. 2, S2). At the same time, the magnetic properties (MS, ARM, SIRM; Fig. 2, S2) all suggest a lower amount of basaltic derived (titanomagnetite) material in the lowermost part of the core.

### Stable isotopes

**Figure 4:**
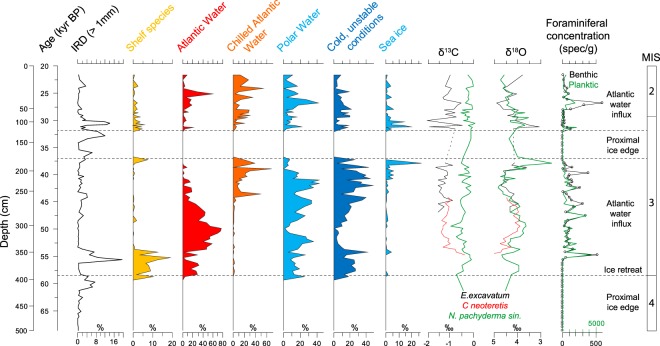
IRD, Stable isotope and foraminiferal distribution from core TTR13-AT-479G. Benthic foraminifera are grouped according to environmental preference; calculated as percentage of the total benthic foraminiferal community. Shelf species: *Elphdium albiumbilicatum, Elphidium hallandense, Elphidium tumidum* and *Buccella tenerimma*^46,53,54,69^; Atlantic species: *Cassidulina neoteretis*^45^*, Cibicides wuellerstorfi*, Miliolida; Chilled Atlantic water indicators: *Islandiella norcrossi*^46,47^; Polar water species: *Cassidulina reniforme*^69,70^ can also tolerate chilled Atlantic water; Cold, unstable conditions: *Elphidium clavatum*^69,70^. Sea-ice indicators: *Islandiella helenae*, *Stainforthia feylin*g*i*^71,72^. Planktic stable isotope values were measured on left-coiled specimens of *Neogloboquadrina pachyderma*. Benthic stable isotopes measurements were carried out on *Elphidium clavatum* for the top part and *Cassidulina neoteretis* for the lower part of the core. Values of *E. clavatum* were standardized to *C. neoteretis* by subtracting 0.02‰ from oxygen isotope value of *E. excavatum* and adding 1.14‰ to carbon isotope values of *E. clavatum*. Planktic stable isotopes were measured on left-coiled *Neogloboquadrina pachyderma*. Foraminiferal concentrations for planktic and benthic foraminifera are shown as number of foraminifera/gram sediment.

The benthic and planktic foraminiferal δ^18^O records of core 479 G (Fig. 4) show relatively little variability, albeit with generally heavier values below about 320 cm (before ca. 54 kyr BP, see age model below), from 200–250 cm (ca. 43–39 kyr BP) and finally from 70 cm (29 kyr BP) till the top of the core date to ca. 21 kyr BP. Unstable isotopic conditions with some very light (<4.0‰) excursions are seen between 195 and 180 cm, and again at 120 cm. No data are available for the bottom of the core, which is near-barren of foraminifera. The δ^13^C record (Fig. 4) displays a generally fluctuating pattern, however with a dominance of negative values in the lower part of the core below ca. 250 cm depth (before ca 45 kyr BP). Although δ^13^C variations cannot simply be explained by changes in single processes such as productivity and ventilation variability, low δ^13^C values at sites close to a glaciated shelf will often reflect melt water release from continental ice masses^26^.

### Age model

Calibrated AMS ^14^C datings of foraminiferal samples (ΔR = 140 ± 30 ^14^C years) provide a firm chronology in the upper about 130 cm of core 479 G (Figs 2,3, Table S1); for details see Supplementary Material. Even taking reservoir age uncertainties and the potentially higher-than-present reservoir effect during glacial times^27–29^ into consideration, our chronology clearly shows that surface sediments at this location are of glacial origin (LGM) with no late glacial to Holocene sediments preserved. As the core top did not show any substantial loss of sediment during coring, this suggests that Holocene erosion or non-deposition has apparently has been typical for this coring site, likely due to high bottom-water energy.

Below 130 cm in core 479 G radiocarbon dates are indefinite and from here on we base our age model on climate and isotope event stratigraphy. Due to the influence of local meltwater as well as lower-salinity polar water entrained by the WGC, a simple correlation of stable isotope data to the marine isotope stratigraphy^30,31^ is not possible. Thus, we depend on a range of data, i.e. stable isotope data (Fig. 4), lithological data (incl. IRD and elemental composition) (Fig. 2) and foraminiferal assemblages (see below, Fig. 4, S4) when extending the chronology beyond the AMS ^14^C measurement range.

Heinrich (H) Event^32,33^ 5a is known for its high detrital carbonate content^34^, while H6 has previously been noted for its low detrital carbonate content in the southeastern Labrador Sea^35,36^. These characteristics imply a correlation of the prominent IRD peak at 360 cm to H5a and the minor detrital carbonate IRD peak at 415–395 cm core depth with H6 (ca. 60 kyr BP^33^). Consequently a MIS 4 depositional environment is inferred for the bottom of the core, which is barren of foraminifera (Fig. 2), suggesting extremely harsh glacial conditions. We place the MIS 4/3 boundary (ca. 57 kyr BP) at the first common occurrence of benthic foraminifera (398 cm core depth) indicating the onset of a more vigorous AMOC (see below), in agreement with previous studies of from the North Atlantic region^37–40^. Although the IRD and detrital carbonate maximum at 360 cm may be assigned to H5a^34^, due to an uncertain absolute age of this event, H5a has not been included in the age model (Fig. 3).

In the oxygen isotope record of core 479 G (Fig. 4), the only section with dominantly lighter δ^18^O values is between about 250 and 320 cm core depth for the benthic and 270 and 340 cm core depth for the planktic foraminiferal isotopes. The latter core segment is further characterised by benthic foraminiferal fauna assemblages indicative of maximum Atlantic water influence (see below; Fig. 4, S4). This interval may thus correspond to the prolonged period of interstadial conditions during Greenland Interstadial (GI) 14, as indicated by the oxygen isotope record of the GRIP ice core^41^. Finally the H3 event (113 cm) may be identified with relative certainty as it is present within the range of ^14^C datings and it has the characteristics of a prominent detrital carbonate content combined with a clear IRD signal (Fig. 2; see Andrews *et al*.^22^).

We refine this chronology using the Si data series from core 479 G, correlating it to the NGRIP ice core, with increased amounts of Si linked to periods of increased Greenland Ice Sheet melting (interstadial periods). This allows us to identify the periods GI14 to GI08 in our data set and to use the chronology of Andersen *et al*.^42^ and Svensson *et al*.^43,44^ to help build our age model (Fig. 2). Consequently, IRD peaks at about 185 cm and 280 cm can be assigned to H4 and H5, respectively (Figs 2,3, S1, Table S2).

### Foraminiferal assemblages and environmental development

The near-absence of foraminifera below 398 cm (Fig. 4, S4) suggests an extremely harsh hydrographic environment prior to the MIS3/4 boundary. Sedimentary properties suggest a high turbidity and high sediment accumulation rates making the area almost inhabitable for foraminifera; such an environment occurs typically close to a glacier front. The onset of MIS 3 at 57 kyr BP, was marked by the immediate development of a foraminiferal fauna representative of an environment with widespread Polar Water mixed with inflow of subsurface water of Atlantic origin (*Cassidulina neoteretis*^45^) (Fig. 4, S4). Sedimentation rates were likely still high as shown by the low foraminiferal concentrations and the presence of allochtonous shelf species in the very early part of MIS 3 between H6 and GS14. At the same time the IRD input (398–350 cm; Fig. 4, S4) suggests some iceberg drift. These data imply a seasonally ice-free ocean, most likely still with extensive winter sea-ice formation and brine formation over the shelf, the latter being responsible for down-slope transport of the foraminiferal shelf species and a low concentration of *in situ* foraminifera.

At ca 54 ka BP (350 cm, onset of GI14) the site experienced a distinct increase in overall abundance of both planktic and benthic foraminifera, and in benthic species associated with subsurface Atlantic water masses and high food availability occurred (Fig. 4, S4). Combined with a near-absence of IRD (Fig. 2), this demonstrates a significant influx of warmer, high-salinity bottom water. By the end of this period the high productivity may have caused a decrease in bottom-water oxygenation, as seen in the relative increase low-oxygen tolerating species at ca. 51 ka BP (310 cm; Fig. 4, S4). The low planktic δ^13^C values support a high primary productivity (Fig. 4).

Warmer conditions with prevailingly subsurface Atlantic water masses persisted until ca. 45 kyr BP (ca. 270 cm), when the influx of Atlantic water gradually decreased and colder, more Arctic conditions characterised by Polar Water are observed (Fig. 4). However, the presence of *Islandiella norcrossi* (Fig. S4), a species, which is often linked to chilled Atlantic water or conditions with mixed Arctic and Atlantic water^46,47^, confirms the continued influx of Atlantic-sourced water as a subsurface current, albeit at lower rates. Sea ice started expanding at ca. 41 kyr BP (Fig. 4), but according to foraminiferal concentrations and δ^13^C values, productivity was decreasing but still relatively high until approx. 40 kyr BP (190 cm).

**Figure 5:**
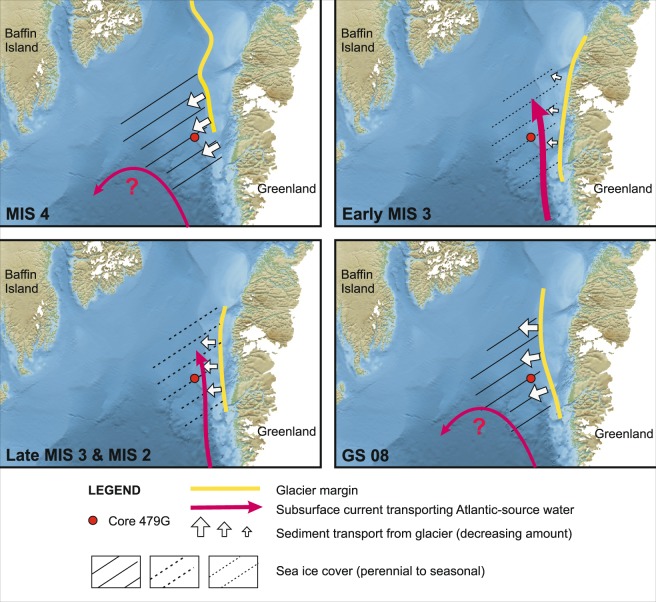
Environmental development off SW Greenland from MIS 4 to MIS 2. Background map shows the present-day glacier extent as comparison. Glacier limit for MIS 4 is based on the position of the Hellefisk Moraine^15,18^, for MIS 2 it is based on Funder *et al*.^15^, while for early MIS 3 and the cooling period of GS08 is an interpretation based on the core 479 G data. Note that the glacier limit reconstruction only shows the part that is inferred from the present data, no other marine sediment core data are yet available for the regions to the north and south and suggested glacier limits are tentative.

From 37–33 kyr BP (170–140 cm) conditions again became harsh for foraminifera, resulting in their near-absence, possibly due to extreme sea-ice conditions and extensive sediment supply from expanding glacier ice. Return of the, albeit more limited, influence of subsurface Atlantic-sourced water masses shortly after 33 kyr BP suggest a change to more open water conditions (140 cm core depth, Figs 2–5, S4), but still with fairly extensive seasonal sea ice conditions (Fig. 4). Between ca. 30 kyr BP (ca. 100 cm) and the LGM (top of core), influence of both Atlantic and Arctic water masses suggest highly variable hydrographic conditions of the subsurface water masses near the site, possibly associated with ocean front movements. From the low IRD percentage and presence of sea-ice indicator species, we may conclude that cold, Arctic conditions favouring low iceberg melting rates prevailed in upper water masses. However, the influx of warm Atlantic-sourced subsurface water was nevertheless still quite significant during MIS 2 until at least 21.5 kyr BP.

## Discussion

Evidence has been provided for large variability of water mass formation and distribution in the subpolar western North Atlantic during the late Quaternary^48^. It has been demonstrated that under glacial climate conditions and during deglaciations, and in particular during large-scale ice-rafting (Heinrich) events, significant changes have occurred in Labrador Sea circulation^36^. The latter study reports enhanced advection of relatively warm intermediate water into the southeastern Labrador Sea during Heinrich events, whereas isotope data and benthic faunal distributions suggest inflow of subsurface Atlantic-derived water into the deep Labrador Sea basin between the Heinrich events^36^. Further indications for a possible link between changes in advection of warm (Atlantic) water and regional ice-rafting in the Davis Strait region have been found by Andrews *et al*.^22^, which supports the scenario of enhanced subsurface ocean heat transport leading to glacial ice sheet destabilization and subsequent large-scale ice-rafting proposed by Moros *et al*.^49^, and also suggested by other studies offshore Greenland^50^. Although IRD events in the Baffin Bay – Labrador Sea may not always be directly correlated to the Heinrich events of the North Atlantic^22^, several other sediment core records from the Labrador Sea have revealed all major Heinrich events and several Dansgaard-Oeschger cycles^34,36,51^.

### MIS 4–3

Extreme environmental conditions are suggested by the absence of foraminifera at the bottom of the core. This interval is also characterised by the presence of fine gravel and some larger-sized IRD as well as elevated XRF values for Si and K (Fig. 2, S2), which are typical elements representative of the continental, siliceous rocks found widespread along the coast of SW Greenland^52^. According to the sediment lithology and structure, it is not a till, but was likely deposited relatively close to, but not right next to, a fairly stable glacier margin. This interval was immediately followed by the major IRD event of H6, which suggests that immediately prior to MIS 3, i.e. during MIS 4, the SW Greenland Ice Sheet had extended far to the southwest and a major break-up of the ice sheet occurred during H6 and at the MIS4/3 boundary (Fig. 5). This extension is also supported by the presence of *Elphidium tumidum* (Fig. S4) in the beginning of MIS 3, which may indicate reworking of older, MIS 5e-5a sediments^cf.53,54^ during deglacial flooding.

Heinrich 6 and the subsequent interval of reworked inner shelf foraminifera (ca. 60–54 kyr BP) may signal a more general collapse of the Greenland Ice Sheet margins at the MIS 4 termination, which at the beginning of MIS 3 contributed to widespread IRD deposition in the Arctic Ocean^7^. In addition, the low ARM and SIRM values (Fig. S2) indicate a relatively low input of magnetic minerals (most likely titanomagnetite) from basaltic sources during MIS 4 and the period around H6. This interpretation implies that during MIS 4, the East Greenland Current entrainment of icebergs from the basaltic provinces of East Greenland and Iceland towards the north-eastern Labrador Sea had ceased. This again may be ascribed to the presence of more permanent sea ice further offshore, most likely in combination with an extensive ice shelf off SW Greenland as suggested by the XRF Si and K records. Within this context it should further be noted that the large Ca peak at ca. 360 cm depth of core 479 G (Fig. 2), assigned to H5a, may also be linked to a large-scale collapse of the NE Canadian MIS 4 ice sheet. Detrital carbonate IRD can typically be related to icebergs produced by glacier calving in the Hudson Bay or Ellesmere Island region, where sedimentary carbonate rocks are widespread.

This ice sheet collapse was immediately followed by a period of very strong influx of Atlantic-sourced subsurface water during the early MIS 3, while the foraminiferal inner shelf species record (Fig. 4, S4) suggests a continued transport of shelf species entrained by (hyperpycnal) melt water flow from the melting ice sheet. This is also supported by the relatively low δ^18^O and δ^13^C values in the early MIS 3 (Fig. 4), which demonstrate that melt water influx was fairly high and suggest large-scale degradation of the (Greenland) ice sheet, albeit with major iceberg release limited to IRD events.

Following the extensive advection of these warmer (ISW) water masses by the WGC in the early MIS 3, likely linked to a strong AMOC seen in the North Atlantic region in particular during the early part of MIS 3^40^, a long-term trend of decreasing warmer water influx and increasing low-salinity Polar Water influx can be observed from 45 kyr BP (270 cm). This is in accordance with the general cooling also recognised in the Greenland ice core records (Fig. 2), where the later part of MIS 3 is characterised by colder stadials and shorter and less pronounced interstadials^41,44^. This finally led to another severe glacial episode with perennial sea ice off SW Greenland (Fig. 5) from ca 37–33 kyr BP (170–140 cm, Greenland Stadial 08, GS08), which may coincide with a generally decreased AMOC regime dominating the North Atlantic region observed during GS08^38^. Just as for the MIS 4 termination, the latter glacial conditions during late MIS 3 ended with a major collapse of adjacent continental ice caps and widespread IRD (H4) deposition, as also recorded in core 479 G. Thus, for the various stages of the Weichselian glaciation we found evidence for a gradual change in glacial Northwest Atlantic circulation patterns and in severity of southwest Greenland glaciations.

### MIS 2

The foraminiferal record of core 479 G (Fig. 4, S4) confirms the presence of Atlantic (Irminger Sea) source water masses during the LGM and the period immediately before (Fig. 5). Peak abundance in Atlantic species near 25 kyr BP, i.e. at the end of MIS 3, suggest a short-lived intensification of the WGC, which may be linked to a larger-scale North AMOC intensification just prior to the LGM as also suggested by an episode of stronger overflow through the Faroe-Shetland Channel at the end of MIS 3^37^.

The transport of foraminiferal shelf species to the deeper-water surroundings of the 479 G core site was more or less continuous during the latter part of MIS 3 and LGM (Fig. 4), which suggests significant brine water transport due to large-scale sea ice formation over the SW Greenland shelf. This conclusion is supported by nannofossil studies of Rahman and de Vernal^55^, who found evidence of seasonally ice-covered surface conditions prevailing in the north-eastern Labrador Sea between 31,000 and 12,600 BP. Enhanced thermohaline circulation in the Labrador-Irminger Sea basin was already reported by Fagel *et al*.^56^ to have started with an increasing Deep Western Boundary Undercurrent activity from the southwest Greenland rise immediately after the LGM. This scenario is supported by a study of magnetic grain sizes in a core from Eirík Drift south of Greenland, which shows enhanced bottom-water circulation peaking shortly after 19,000 cal yrs BP^57^.

Enhanced circulation of (warmer) subsurface ISW since the LGM may have played an important role for the deglacial retreat of the southwestern Greenland Ice Sheet dated as early as about 18,000 cal. yrs BP^58^, also supported by the findings of Sheldon *et al*.^11^ and Jennings *et al*.^12^, who found a very early onset of shelf ice retreat and immediate influx of Atlantic-sourced water off Uummannaq, NW Greenland, after the LGM. Thus various evidences suggests that the LGM oceanographic conditions over the Southwest Greenland outer shelf are typically dominated by a persisting WGC also during glacial conditions, with extensive seasonal sea ice formation and an only moderate GIS (mid?) shelf advance. Brine formation due to seasonal freezing would have been an important process that promoted advection of warm Atlantic (subsurface) water masses into the north-western Atlantic^59^. Moreover, benthic foraminiferal fauna studies by Rasmussen *et al*.^36^ indicate advection of Atlantic-derived water masses into the north-eastern Labrador Sea not only between Heinrich events, but also during them.

### SW Greenland ice sheet extent

We may conclude that in course of the last glacial cycle, the West Greenland ice sheet most likely reached a maximum extent on the shelf not during MIS 2, but in fact during MIS 4 (Fig. 5). Little is known about the MIS 4 configuration and extent of the GIS, but in the Arctic, MIS 4 also coincided with extreme outer shelf glaciation on the eastern Siberian shelf^6,7^ as well as with extensive land-ward ice extent in Siberia^60^. Apart from the bottom section of core 479 G representing extreme glacial conditions during MIS 4, another period characterised by glacial conditions offshore Southwest Greenland that was more severe than under the LGM is noted for the core record around 170–140 m subbottom depth (Fig. 4). This interval near-barren of foraminifera (Fig. 2) may likely be assigned to the cooling period of immediately following GI08 (i.e. GS08)^40,41,44^.

Previous studies of southwest Greenland shelf glaciations report a total of only five extreme shelf edge glaciations over the last ca 4.5 million years, of which the youngest is assigned to the MIS6 Saale glaciation^61^. This is supported by evidence of extremely deep-draft (ca 900 m) icebergs drifting within the North Atlantic subpolar gyre, presumably originating from a southern Greenland ice shelf calving at, or beyond, the continental shelf edge^62^, i.e. seaward of the Outer Hellefisk Moraines. Thus, a decreasing trend in southwest Greenland shelf glaciation may be observed since MIS6. Our findings are further thought to have implications for the possible age of origin of the Outer Hellefisk Moraines, which according to our results may be attributed to the MIS4 shelf glaciation rather than to the MIS2 (LGM) glacial stage, at least for the southern Davis Strait region.

The difference between the MIS4 and MIS2 glaciation can be explained by gradually increasing Atlantic-derived (ISW) water mass advection and WGC activity in the course of the last glaciation, and may reflect a fundamental change in the AMOC and the North Atlantic circulation pattern since the last interglacial (MIS 5e), when deep-water formation in the Labrador Sea was found to be absent^48^. Such a shift in North Atlantic ocean heat transport may have been accompanied by a corresponding decrease of warm-water transport by the Norwegian Atlantic Current, and may be an important factor, when explaining differences in the extent of maximum glaciation between NW Europe and Scandinavia and SW Greenland, when comparing MIS 2 and 4. When comparing the difference of the MIS 2 and MIS 4 southwest Greenland shelf glaciations, another important factor may have been the difference in North American ice sheet size and location during MIS 2 and MIS 4^3^ as well as their respective effect on atmospheric circulation. For the much larger MIS2 ice sheet, atmospheric modelling studies^63^ indicate a regional decrease in precipitation over the southwest Greenland region, which should imply lower snow accumulation rates over SW Greenland and consequently a decreasing glacier growth and ice sheet shelf expansion. Further to the north in the Baffin Bay region, the Greenland Ice Sheet had also significantly expanded during MIS4, but here the MIS 2 glaciation seems to have been more extensive^13,14,64^. It should be noted that data are yet insufficient to evaluate whether the entire Outer Hellefisk Moraines complex extending further north is in fact of the same age as found for the area of our study. Nevertheless, we tentatively conclude that the relatively weak influx of Atlantic-sourced water during MIS 4 may have allowed the GIS to extent relatively far to the shelf edge also in surrounding regions. This in contrast to MIS 2, when our data point to a stronger Atlantic water flux.

## Conclusions

Based on the multiproxy analyses of a marine sediment core from the northernmost Labrador Sea, we here present new evidence of a significant extension of the Greenland Ice sheet (GIS) in the early Weichselian (MIS 4) in the Davis Strait region. Our results suggest that the ice sheet expanded further onto the West Greenland shelf than during the Last Glacial Maximum (LGM, MIS 2), and it retreated fairly abruptly at the onset of early MIS 3 warming. During MIS 4 the GIS ice margin reached close to the shelf edge, but did not exceed the shelf edge during the time period covered in our study (>60,000 years). The nearby sector of the Outer Hellefisk Moraine is thus likely of MIS 4 age.

During much of the last 60 kyr (starting in early MIS 3) Atlantic-sourced water has penetrated into the Labrador Sea at intermediate depths, although the strength of Atlantic-water influx has varied significantly over time and was clearly weaker during the second part of MIS 3 and during MIS 2 than during the vigorous AMOC of the early MIS 3. One additional larger ice sheet expansion likely occurred between just after Greenland Interstadial (GI) GI08 (ca. 37–33 kyr BP). During our >60 kyr long record, there is thus a strong inverse relationship between the advection of the relatively warm Atlandic-sourced water to the West Greenland margin and the shelf-ward expansion of the GIS. This result highlights not only that regional ice sheet extent may well be uncoupled from the global ice volume, but also illustrates the fast response of the Greenland Ice Sheet to climate and ocean change.

## Methods

Nine ^14^C-datings from the top 130 cm of core 479 G were performed at the Aarhus AMS ^14^C Dating Centre, Aarhus University, and the Leibniz Laboratory for Radiometric Dating and Stable Isotope Research, Kiel University (Table S1), using monospecific planktic foraminifera of *Neogloboquadrina pachyderma* (left-coiled). One sample of mixed benthic foraminifera was also dated (Fig. 3, Table S1), but not included in the age model. We used the OxCal 4.2 software^65^ and the Marine13 calibration curve^66^ and a ΔR = 140 ± 35 (see Supplementary material) in the age model for this top section of the core (Fig. 3). For the lower part of the core, we correlated the Si (silica) records to the Dansgaard-Oeschger event stratigraphy of Andersen *et al*.^42^ and Svensson *et al*.^43,44^, supported by Heinrich event stratigraphy^33^ and a correlation to marine isotope stages^30,31^) (Figs 2,3). For further details, see Supplementary material.

Multi-element geochemical data (Fig. 2, S2) was obtained with the Avaatech X-ray fluorescence (XRF) core scanning system^24^ at the Royal Netherlands Institute for Sea Research (NIOZ), Texel at a 1-cm resolution.

Magnetic measurements were made at Lund University, Sweden, on sediment subsamples packed into cubes with 2.2 cm dimensions. Magnetic susceptibility (MS) was measured using a Geofyzica Brno (now Agico) KLY2 kappabridge. Anhysteretic remanent magnetization (ARM) was induced in each sample with a Molspin alternating field demagnetiser, using a direct current (DC) bias field of 0.1 milliTesla (mT) that was superimposed on a peak AF of 100 mT. Saturation Isothermal Remanent Magnetization (SIRM) was induced in a field of 1 T by a Redcliffe 700 BSM pulse magnetizer. The induced remanences (ARM and SIRM) were measured with a Molspin Minispin (Fig. 2, S2). The MS and remanence parameters were corrected for the dry mass to provide mass specific units.

Grain-size analyses were carried out through wet-sieving of the sediment through sieves with mesh sizes of 0.063, 0.1 and 1.0 mm as part of the foraminiferal laboratory analyses. The IRD-content was calculated as the weight % of the sediment fraction > 1 mm, thus mainly identifying the larger IRD grains (Fig. 2). Choice of this relatively large grain size for IRD ensures that the signal is not subject to noise from foraminiferal abundances or changes in current strength. Tests of the validity of this proxy through comparison to the 0.1–1.0 mm sediment fraction showed that in the sections of the core with very few foraminifera, also the 0.1–1.0 mm fraction peaked during IRD peaks (Fig. S2). In sections with abundant foraminifera, this pattern is obscured. Gravel and larger rock fragments were not sampled and are thus not included in the particle size data but the content of larger grains was noted in the core description.

For foraminiferal analyses (Fig. 4; Fig. S4), the samples were dried, weighted, and washed through sieves with mesh sizes of 0.063, 0.1 and 1.0 mm and foraminifera were counted from the 0.1–1.0 mm fraction. The 0.063 mm fraction was checked for additional species, none were found. Where possible at least 300 benthic and 300 planktic individuals were counted in each sample. However, due to low foraminiferal concentrations in selected intervals (Fig. S3), this was not always possible and therefore all samples with more than 40 specimens were included in the calculations.

Oxygen and carbon isotope measurements of the planktic species *Neogloboquadrina pachyderma* (sinistral) Ehrenberg and benthic species *Cassidulina neoteretis* Seidenkrantz and *Elphidium clavatum* Cushman (Fig. 4) were performed on a Finnigan MAT252 mass spectrometer at Woods Hole Oceanographic Institution (WHOI) following the procedure described by Ostermann and Curry^67^. An overlap between the benthic isotopes measured on the two species, made it possible to create a combined benthic stable isotope curve. Values of *E. clavatum* were standardised to *C. neoteretis* by subtracting 0.02‰ from oxygen isotope and adding 1.14‰ to carbon isotope values of *E. clavatum*. All values are calibrated to the PDB scale.

## Supplementary information

Supplementary material

Supplementary data 1

## Data Availability

All published data are included in supplementary material.
