# Supplementary material for: Southwest Greenland shelf glaciation during MIS 4 more extensive than during the Last Glacial Maximum
Source: Sci Rep. 2019 Oct 30;9:15617. doi: 10.1038/s41598-019-51983-3 (PMC6821744; doi:10.1038/s41598-019-51983-3)
Supplement: Supplementary file 1 — Supplementary material [file 41598_2019_51983_MOESM1_ESM.pdf]

Supplementary material to

Southwest Greenland shelf glaciation during MIS 4 more extensive than during the Last Glacial Maximum

Marit-Solveig Seidenkrantz, Antoon Kuijpers, Jesper Olsen, Christof Pearce, Sofia Lindblom, Johan Ploug, Piotr Przybyło, Ian Snowball

Content

The below supplementary material provide additional information but also expanded discussions.

- Sediment description of core TTR13-AT-479G
- Elemental composition and magnetic properties
- Sediment core chronology (expanded)
- Foraminiferal assemblages and environmental development (expanded)
- References

Sediment description of core TTR13-AT-479G

Gravity core TTR-13-AT-479G (hereafter 479G) is located at 1033 m water depth in the south-eastern region of the Davis Strait (64°24.37'N, 54°45.08'W, 503 cm long; Kenyon et al., 2004). The sediment (Fig. S1) primarily consist of olive-green to olive-grey (mainly 5GY4/1 on the Munsell colour system) clay with silt and fine sand interrupted by layers of sand and gravel clearly visible to the naked eye at 100-113 cm, 158-163 cm, 353-357 cm, 360-364 cm, 368-369 cm and 403-413 cm.

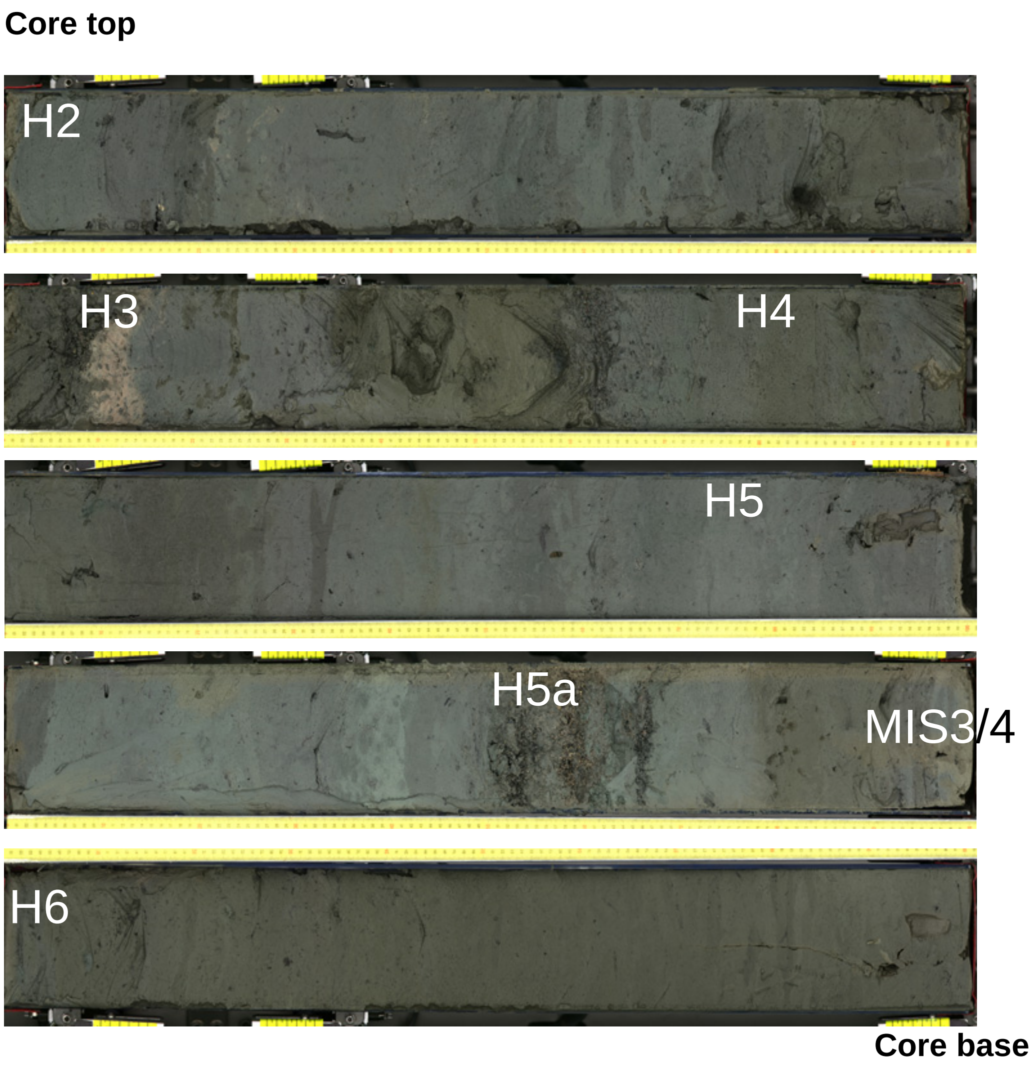

Figure S1. *Line scan photograph of sediment core TTR13-AT-479G from Avaatech core scanning. Each core section is ~1 m long, with a total core length of 503 cm. The portion of sediment deposited during Heinrich (H) events H2-H6 as well as the approximate location of the Marine Isotope Stage (MIS) boundary between MIS3 and MIS 4 are indicated.*

Additional layers with larger grains are visible in the grain size data (grains >1 mm; Fig. 2, S1), particularly at 101-113 cm, 135-145 cm, 276-279 cm and 398-413 cm. The uppermost layer includes a sublayer (108-113 cm) that has a clear reddish-pink colour (10YR5/1). A thin lens of reddish colour is also seen at ca 21-22 cm, while others are only weakly reddish in colour (353-369 cm interval). From ca. 400-503 cm (bottom of core) the sediment contains a larger fraction of small gravel and a few stones in an otherwise homogeneous clay matrix; these are however not evident in the grain size fraction, as grain size analyses were carried out on the more fine-grained matrix, excluding stones and larger pebbles. A larger content of gravel is seen between ca 402-417 cm.

Elemental composition and magnetic properties

Coarser IRD (> 1 mm) is found concentrated in several peaks, some of which coincide with a maximum in the XRF Ca (and Ca/Sr) records (Fig. 2, Fig. S2) and lower concentrations of magnetic minerals. In general, there is an inverse relationship between IRD and the magnetic concentration parameters (χ, ARM and SIRM; Fig. S2). The 0.1-1.0 mm fraction consists of both mineral grains (incl. IRD) and foraminifera. A marked feature in the record for the three elements K, Fe and Ti is the occurrence of a pronounced negative excursion at about 1.10 m and 3.60 m depth, which coincides with the well-defined peak in the Ca counts.

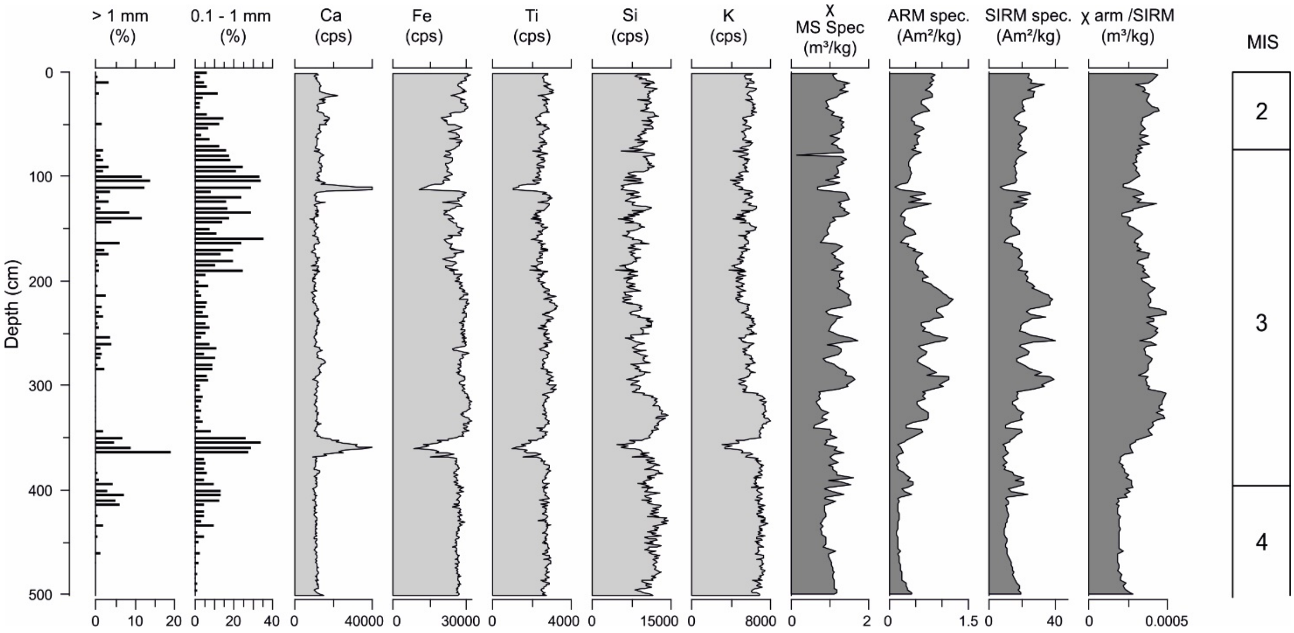

Figure S2. *Fraction of grains >1 mm ((used as indicator of ice-rafted debris (IRD), and 0.1-1.0 mm as percentage of the total sediment, elemental composition (cps = coups per second) and magnetic parameters (*χ = specific magnetic susceptibility) *of core 479G plotted versus depth. MIS = Marine Isotope Stage.*

Sediment core chronology

Nine AMS ^{14}C-datings were performed for core 479G at the Aarhus AMS ^{14}C Dating Centre (AARAMS), Aarhus University (AAR, Table S1), and the Leibniz Laboratory for Radiometric Dating and Stable Isotope Research, Kiel University (KIA) (Table S1; Figs. 2, 3), using the planktonic foraminifera *Neogloboquadrina pachyderma* (sinistral, left-coiled). Four dates on planktonic foraminifera were used in the age model, while one date showed reworking of older material (outlier) and dates below 130 cm were indefinite (Table S1). Further, one sample of mixed benthic foraminifera was also dated in core 479G (Fig. 3). We used the depositional model option in the OxCal 4.2 software (Bronk Ramsey, 2009) and the Marine13 calibration curve (Reimer et al., 2013) to establish the age models for the upper part of core 479G (Fig. 3).

Table S1. *List of ^{14}C dates for cores TTR13-AT-479G. Reservoir age : 140±35 years.*

Lab.code	Core depth (cm)	Material dated	Age (^{14}C yr BP)	Calibrated age range (cal yr BP, 2σ)	Model age (cal yr BP)
AAR-10223	0-1	*N. pachyderma* sin	18,330±100	21191 - 21856	21583
AAR-10240	44-46	*N. pachyderma sin*	23,380±170	26676 - 27504	27213
KIA 34381	50-52	Mixed benthic foraminifera	23,780±130	26404 - 25778	n/a
KIA 34382	50-52	*N. pachyderma* sin	24,510±130	27727 - 28358	27955
AAR-10241	94-96	*N. pachyderma* sin	36,550±750	39009 - 41894	n/a
KIA 34383	130-132	*N. pachyderma* sin	28,200±210	31086 - 31882	31607
KIA 34384	190-192	*N. pachyderma* sin	>52,090	n/a	n/a
KIA 34385	200-201	*N. pachyderma* sin	>53,600	n/a	n/a
AAR-10224	264-266	*N. pachyderma* sin	>49,000	n/a	n/a
AAR-10225	364-366	*N. pachyderma* sin	>50,000	n/a	n/a

The reservoir correction of the Davis Strait region is unknown; in coastal regions off West Greenland data are very variable with an average ΔR~135±65 (9 closest sites with a distance <800 km; see Reimer and Reimer, 2001). We are aware that the local reservoir age may have varied over time in concert with oceanic circulation changes and sea ice coverage, and significantly larger reservoir ages have in fact been suggested for the glacial North Atlantic (e.g., Bard et al., 1994; Thornalley et al., 2011; Stern and Lisiecki, 2013). However, the one date based on benthic foraminifera suggests a slightly younger age than the date based on planktonic foraminifera from the same depth, indicating strong ventilation of the water column. This would suggest that the glacial reservoir age at our study

site may in fact have been close to present-day values and as no data are yet insufficient to establish a proper time dependent ΔR variability, we have here used the ΔR=140±35 in accordance with previous studies from Disko Bugt (Lloyd et al. 2011; Sheldon et al., 2016; Jennings et al., 2017). This is also supported by the high Atlantic-sourced subsurface water influx suggested by the benthic foraminiferal assemblages.

For extending the chronology beyond the AMS ^{14}C measurement range, we depend on interpretation of the XRF core scanning data (Fig. 2), lithological data (IRD) (Fig. 2), stable isotope data (Fig. 4) and foraminiferal assemblages (Fig. 4, Fig. S4), which may be compared with results from previous studies in the Arctic and northern North Atlantic. Due to the relatively shallow water depth of the site, which today is influenced by local melt water and both Polar surface water and Atlantic subsurface water masses entrained by the West Greenland Current (Fig. 1A), combined with the relatively short time span covered by the core with lack of the distinct isotopic excursions of Marine Isotope Stage (MIS) 1 and MIS 5, a precise correlation to the marine isotope chronology (Martinson et al., 1987; Lisiecki and Raymo, 2005) is not possible.

Although IRD events in the Baffin Bay–Labrador Sea may not always be directly correlated to the Heinrich (H) events of the North Atlantic (see Andrews et al., 1998) several previous studies from the Labrador Sea have revealed all major Heinrich events and several Dansgaard-Oeschger cycles (Weber et al., 2001; Rashid et al., 2003). In core 479G, H3 may be identified with reasonable certainty to 113 cm as it is located within the range of ^{14}C dating. The correlation of this IRD event to H3 is supported by the high amount of detrital carbonate event seen in this interval, as a high detrital carbonate content is common for H3 (see also Andrews et al., 1998). Furthermore, the distinct signal of high IRD (seen clearly both in the >1 mm and in the 0.1-1.0 mm fractions) and a clear detrital carbonate maximum at 360 cm allows this event to be assigned to H5a (Rashid et al., 2003; corresponding to Greenland Stadial 15; Svensson et al., 2008).

In contrast, H6 is known to have low concentrations of detrital carbonate in the southeastern Labrador Sea (Stoner et al., 1996; Rasmussen et al., 2003). Supported by its stratigraphical position below H5a in core 479G, the low detrital carbonate content of the IRD event at 415-395 cm core depth implies a likely correlation with H6 for this IRD peak. Although it cannot be fully ruled out that this lowermost IRD peak is not directly equivalent to H6, but corresponds to a period of increased local iceberg release from Greenland, the age of such a melting event would nevertheless approximately correspond to H6.

The sediments below this lowermost IRD event are allocated to MIS 4, a chronology which is supported by the fact that the sediment of this lowermost part of the core is almost barren of foraminifera but contains a larger fraction of gravel and even a few stones. The high sedimentation rates indicated by the lithology and not the least the virtual absence of foraminifera suggests that the entire bottom part of the core down to core base is made out of MIS 4 deposits. The MIS 4/3 boundary is thus placed at the first persistent occurrence of both benthic and planktic foraminifera at 398 cm core depth.

Above 398 cm in the core, the benthic foraminiferal assemblage shows a sudden and significant influx of Atlantic-sourced water (Fig. 4). This indicates the onset of a vigorous Atlantic Meridional Overturning Circulation (AMOC), as has previously been described based on sediment magnetic parameters and microfossils for the North Atlantic during the interstadials of MIS 3 (e.g., Kissel et al., 1999; Rasmussen et al., 2003). Moreover, from the overflow record of the Faeroe region (Kuijpers et al., 1998) it appears that AMOC intensity at the beginning of MIS 3 was comparable with Holocene conditions. Stable oxygen isotope core data from the Denmark Strait (Hagen and Hald, 2002) also support an active AMOC at the beginning of MIS 3.

Table S2. *Estimated age of IRD events used in the age model of core TTR13-AT-479G.*

Event	Depth in core (cm)	Age (yr b2k)	Age (cal yr BP)	Reference
Heinrich 2	11		24000	Hemming, 2004
Heinrich 3	113		ca. 31 000	Hemming, 2004
GI08	182	38 220±724		Andersen et al., 2006
Heinrich 4	185		38 000	Hemming, 2004
GI09	210	40 160±790		Andersen et al., 2006<br>Svendsson et al., 2006
GI10	244	41 460±817		Andersen et al., 2006<br>Svendsson et al., 2006
GI11 onset	251	43 340±868		Andersen et al, 2006;<br>Svensson et al. 2008
GI12 onset	278	46860±956		Andersen et al, 2006;<br>Svensson et al. 2008
Heinrich 5	280		45 000	Hemming, 2004
GI13 onset	290	49 280±1050		Svennson et al 2008
GI14 onset	351	54 220±1150		Svennson et al 2008
Heinrich 5a max	360			Rashid et al. 2003
MIS 3/4 boundary	398	ca. 57 000	58 960	Martinsson et al. 1987;<br>Lisiecki and Raymo, 2005
Heinrich 6	417	ca. 60 000		Hemming. 2004)

The correlation of sediment proxies influenced by melting of surrounding ice sheets linked to Dansgaard-Oeschger cycles has previously been shown from the region (Simon et al. 2014). Based on the overall stratigraphical framework, we were able to correlate the Si data series from core 479G to the NGRIP ice core, with increased amounts of Si linked to periods of increased Greenland Ice Sheet melting (interstadial periods). This link allowed us to identify Greenland Interstadial (GI) periods GI14 to GI08 (Andersen et al. 2006; Svensson et al. 2008) in our data set and to use the chronology of Andersen et al. (2006) and Svensson et al. (2006, 2008) for ages of these GI events. This stratigraphy is supported by the fact that the only longer interval dominated by lighter $\delta^{18}O$ values is at ca. 250-320 cm core depth for the benthic and ca. 270-340 cm core depth for the planktonic isotopes. The interval is further characterised by benthic foraminiferal fauna assemblages indicating maximum Atlantic water influence. Isotope data and foraminiferal assemblages thus support that the interval corresponds to the prolonged period of interstadial conditions during GI14 (ca. 54-50.000 yrs BP) (see Johnsen et al. 2001; Svensson et al., 2008). Consequently, other IRD peaks at about 180 and 280 cm could be assigned to H4 and H5, respectively (Figs. 2, 3, Table S2).

Foraminiferal assemblages and environmental development

The foraminiferal fauna record (Fig. 4; Fig. S4; Table S3) suggests an extremely harsh hydrographic environment for the oldest part of the record below 398 cm (prior to MIS3/4 boundary, 60 kyr BP), with conditions that apparently were unsuitable for the life of both benthic and planktic foraminifera. In this part of the record, very low concentrations of foraminifera (Fig. 4) resulted in so low counts (<40 specimens) that no percentage calculations were possible (Fig. S3). There is no indication of dissolution, and we believe that the low foraminiferal concentrations may be ascribed to high sediment accumulation rates and high turbidity, as also supported by the lithology. This stage was followed by the development of a fauna representative of an environment with widespread Polar Water (*Cassidulina reniforme, Elphidium clavatum*; see e.g. Polyak *et al.,* 2002), some albeit

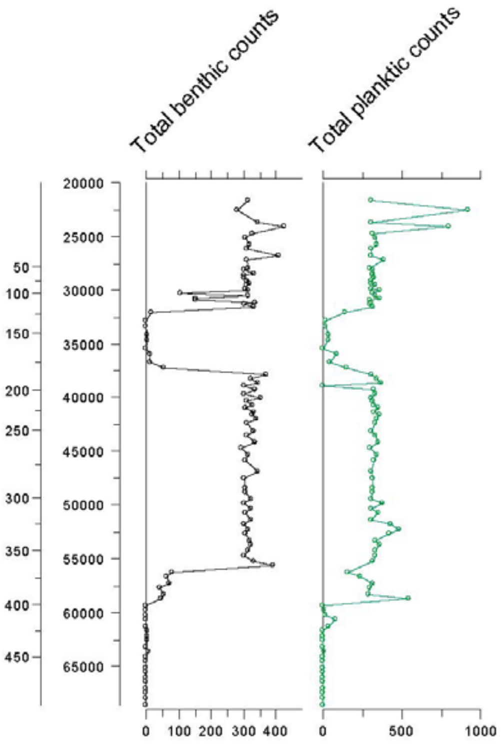

Figure S3. *Total number of benthic and planktic foraminifera analysed in each sample of core 479G. Percentage calculations were only performed when >40 specimens were counted.*

limited inflow of water of Atlantic origin (*Cassidulina neoteretis*; see Seidenkrantz, 1995) and allochtonous influx of shelf species (*Elphidium tumidum*, *Elphidium hallandense, Buccella frigida* and *Buccella tenerimma* (e.g., Rytter *et al.*, 2002; Grøsfjeld *et al.*, 2006) in the very early part of MIS 3 between H6 and GS14.

Some of the shelf foraminifera, in particularly *Elphdium tumidum*, have previously been described from Eemian (MIS 5e) or early Weichselian deposits in the Arctic (Grøsfjeld et al. 2006; Möller et al., 2008) and they may thus be reworked from such older deposits. An actual Eemian age of the sediment itself is highly unlikely, as the foraminifera do not represent a complete assemblage, only some of the largest foraminifera that may tolerate reworking; a complete Eemian assemblage would be much more diverse and with much higher foraminiferal concentrations. We thus believe that these shelf species were deposited at the site by flushing of meltwater from the retreating glacier.

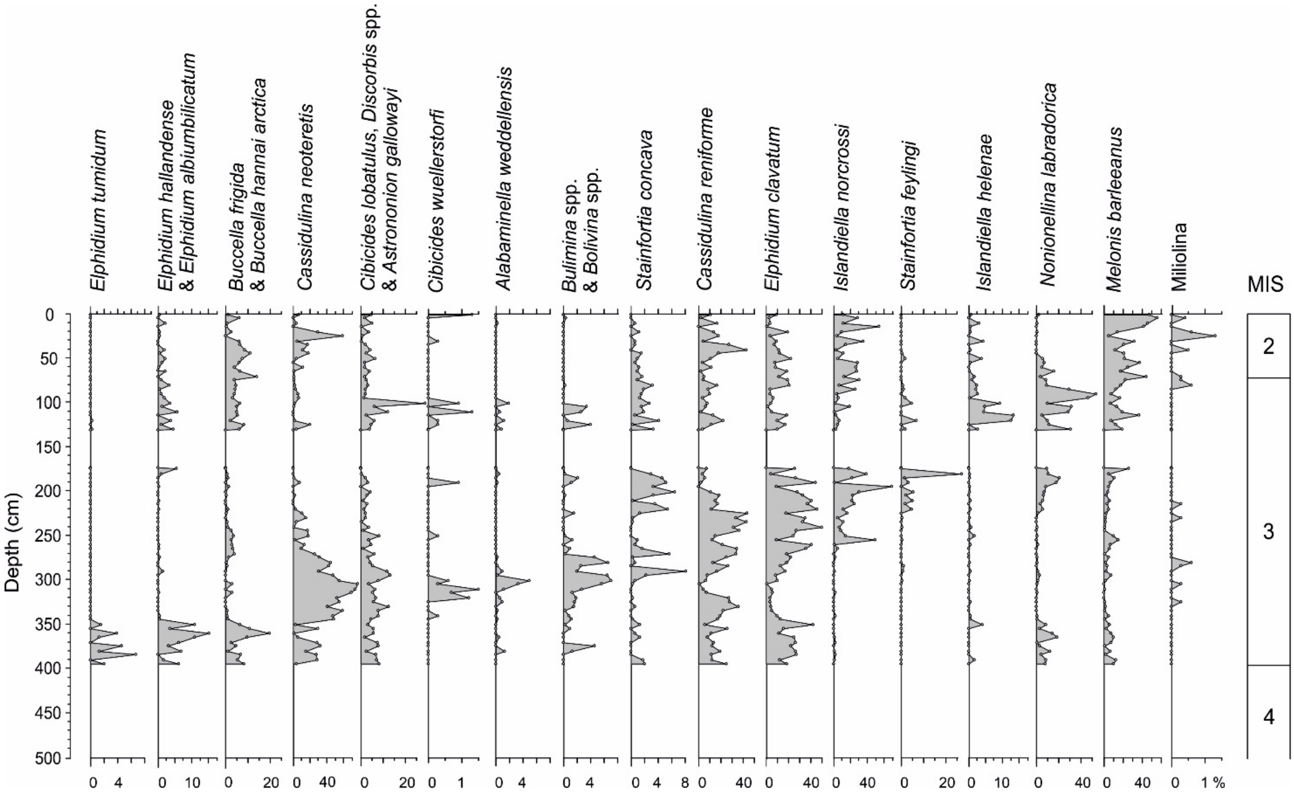

Figure S4. *Benthic foraminiferal assemblage distribution through core TTR13-AT-479G versus depth; data are shown as percentage distribution in relation to the total benthic foraminiferal assemblage. MIS = Marine Isotope Stage.*

Following this initial marine stage, both the planktic and benthic foraminifera increase significantly in abundance, in particular during GI14 (ca. 54-51 kyr BP). The benthic assemblage is dominated by *C. neoteretis*, but also *Cibicides wuellerstorfi* and *Alabaminella weddellensis* (Fig. S4), and indicate a strong influx of Atlantic-sourced water masses and high food availability (see e.g., Schnitker, 1980; Seidenkrantz, 1995; Rasmussen *et al.*, 1996). Combined with the virtual absence of IRD (Figs. 2) this demonstrates a significant influx of warmer and more saline (i.e. Atlantic) water both at the surface and at the sea floor. By the end of this period the high productivity may have caused a decrease in bottom-water oxygenation (e.g. *Bulimina* spp., *Bolivina* spp, *Stainforthia concava;* see e.g. Sen Gupta and Machain-Castillo, 1993) (310-260 cm). The low $\delta^{13}C$ values (Fig. 4) support a high productivity, while the only small difference between benthic and planktic $\delta^{13}C$ ($\Delta\delta^{13}C$) indicate generally good convection and little stratification.

The strong influx of Atlantic water masses persisted for a longer period, after which at ca. 270 cm depth (ca. 45 kyr BP) a change to colder conditions is observed through an increase in Polar Water benthic species (*C. reniforme* and *E. clavatum*). The following interval (until the start of Greenland Stadial GS11, ca 42,000 BP) was characterized by alternating *E. excavatum* and *Islandiella norcrossi*. The latter species has been found related to mixed Polar and Atlantic water north of Iceland (Rytter *et al.,* 2002), and chilled Atlantic water in the Disko Bugt (Lloyd, 2006). This suggests unstable conditions with continued episodic inflow of Atlantic-source water possibly combined with brine formation alternating with melt-water influx. According to the foraminiferal concentrations and $\delta^{13}C$ values, productivity had decreased but was still relatively high in the interval up to app. 190 cm. A significant peak in planktic $\delta^{18}O$ (Fig. 4) and very high frequencies of *Stainforthia feylingi* (Fig. S4), which tolerates high productivity and low bottom-water oxygenation (see Knudsen and Seidenkrantz, 1994; Rytter *et al.,* 2002; Knudsen *et al.,* 2008) often found in sea-ice settings (Seidenkrantz, 2013) at 181 cm (38 kyr BP) may have been caused by a severe melt-water event causing stratification, increased sea ice and reduced bottom-water oxygenation.

The interval at ca. 170-140 m depth (37-33 ka BP) is nearly barren of foraminifera, suggesting that environmental conditions again become harsh for both benthic and planktic foraminiferal fauna. The previous expanding sea ice and presence of IRD suggest that the site was subject to extreme sea-ice conditions and likely also iceberg release. A subsequent reintroduction of the, albeit more limited, influence of (subsurface) Atlantic water masses at 33 kyr BP (140 cm core depth) is indicated by a mixed fauna of Atlantic (*C. neoteretis, Cibicides wuellerstorfi*) and Polar Water (*C. reniforme*) species (Figs. 4, S4) together with increased accumulation of IRD (Fig. 2). *Islandiella helenae* is

found connected to summer ice-edge productivity in areas of seasonal ice cover in the Barents and Kara seas (Steinsund *et al.*, 1994; Polyak *et al.*, 2002; Seidenkrantz, 2013) and its presence together with the bloom of *Nonionellina labradorica* that is often found in connection to ocean fronts (Hald and Steinsund, 1992; Rytter *et al.*, 2002), suggests that the frontal zone between Atlantic and Polar water was found in this region. Between that time (app. 100 cm; ~30 kyr BP) and the Last Glacial Maximum (top of core; ~21 kyr BP) the influence of both Atlantic and Arctic water masses suggests markedly different hydrographic conditions of upper water masses near the site, possibly associated with ocean front movements. This is also indicated by peak abundance of the planktic and benthic fauna observed at ca. 40 cm core depth (26-27 kyr BP). From the low IRD percentage and the presence of *I. helenae*, we may conclude, however, that cold, sea-ice filled Arctic conditions favoring low iceberg melting rates prevailed at the surface, while the persistent occurrence of *C. neoteretis* and *Melonis barleeanus*, which is also often found in connection to Atlantic bottom waters, tell us that the influx of warm Atlantic-sourced subsurface water was nevertheless significant at least during the early part of the LGM (at least until core top at 21.5. kyr BP).

Table S3. *List of benthic foraminiferal species mentioned in text or figures.*

Alabaminella weddellensis (Earland, 1936)	*Elphidium albiumbilicatum* (Weiss, 1954)
Astrononion gallowayi Loeblich and Tappan, 1953	*Elphidium clavatum* Cushman, 1930
Bolivina spp.	*Elphdium hallandense* Brotzen, 1943
*Buccella frigida (*Cushman, 1922)	*Elphdium tumidum* (Gudina, 1969)
Buccella hannai arctic Voloshinova, 1960	*Islandiella helenae* Feyling-Hanssen & Buzas, 1976
Bulimina spp.	*Islandiella norcrossi* (Cushman, 1933)
Cassidulina neoteretis Seidenkrantz, 1995	*Melonis barleeanus* (Williamson, 1858)
Cassidulina reniforme Nørvang, 1945	*Miliolina*
Cibicides lobatulus (Walker & Jacob, 1798)	*Nonionellina labradorica* (Dawson, 1860)
Cibicides wuellerstorfi	*Stainforthia concava* (Höglund, 1947)
Discorbis spp.	*Stainfortia feylingi* Knudsen & Seidenkrantz, 1994

References

Andersen, K. K. *et al.* The Greenland Ice Core Chronology 2005, 15-42 ka. Part 1: constructing the time scale. *Quatern. Sci. Rev.* **25**, 3246-327 (2006).

Andrews, J. T., Kirby, M. E., Aksu, A., Barber, D. C. & Meese, D. Late Quaternary detrital carbonate (DC-) layers in Baffin Bay marine sediments (67°-74°N): correlation with Heinrich events in the North Atlantic? *Quatern. Sci. Rev.* **17**, 1125-1137 (1998).

Bard, E. *et al.* The North Atlantic atmosphere-sea surface 14C gradient during the Younger Dryas climatic event. *Earth Planet. Sci. Lett.* **126** (4), 275–287. doi:10.1016/0012-821X(94)90112-0 (1994).

Bronk Ramsey, C. Bayesian analysis of radiocarbon dates. *Radiocarbon* **51**, 337–360 (2009).

Grøsfjeld, K., Funder, S., Seidenkrantz, M.-S. & Glaister, C., Last Interglacial marine environments in the White Sea region, northwestern Russia. *Boreas* **35**, 493-520 (2006).

Hagen, S. & Hald, M. Variation in surface and deep water circulation in the Denmark Strait, North Atlantic, during marine isotope stages 3 and 2, *Paleoceanography* **17** (4), 10.1029/2001PA000632 (2002).

Hald, M. & Steinsund, P. I. Distribution of surface sediment benthic foraminifera in the southwestern Barents Sea. *J. Foramin. Res.* **22**, 347–362 (1992).

Hemming, S. R. Heinrich events: Massive late Pleistocene detritus layers of the North Atlantic and their global climate imprint. *Rev. Geophysics* **42**, RG1005, 10.1029/2003RG000128 (2004).

Jennings, A. E. *et al.* 2017. Ocean forcing of Ice Sheet retreat in central west Greenland from LGM to the early Holocene. *Earth Planet. Sci. Lett.* **472**, 1–13, 10.1016/j.epsl.2017.05.007.

Johnsen, S. J. et al. Oxygen isotope and palaeotemperature records from six Greenland ice-core stations: Camp Century, Dye-3, GRIP, GISP2, Renland and North GRIP. *J. Quatern. Sci.* **16**, 299-307 (2001).

Kenyon, N.H., Ivanov, M.K., Akhmetzhanov, A.M., Kozlova, E.V., Mazzini, A. (Eds.), Interdisciplinary studies of North Atlantic and Labrador Sea Margin Architecture and Sedimentary Processes. *Intergovernm. Oceanograp. Comm. Technical Series* **68**, UNESCO, 92 pp (2004).

Kissel, C. *et al.* Rapid climatic variations during marine isotopic stage 3: magnetic analysis of sediments from Nordic Seas and North Atlantic, *Earth Planet. Sci. Lett.* **171**, 489-502 (1999).

Knudsen, K.L. & Seidenkrantz, M.-S. *Stainforthia feylingi* new species from arctic to subarctic environments, previously recorded as *Stainforthia schreibersiana* (Czjzek). *Cushman Found. Foramin. Res. Spec. Publ.* **32**, 5-13 (1994).

Knudsen, K. L., Stabell, B., Seidenkrantz, M.-S., Eiríksson, J. & Blake W., Jr. Deglacial and Holocene conditions of the southern Nares Strait, north Baffin Bay: sediments, foraminifera, diatoms and stable isotopes, *Boreas* **37**, 346–376, 10.1111/j.1502-3885.2008.00035.x (2008).

Kuijpers, A. *et al.* Norwegian Sea overflow variability and NE Atlantic surface hydrography during the past 150,000 years, *Mari. Geol.* 152, 75-99 (1998).

Lisiecki, L. E. & Raymo, M. E. A Pliocene-Pleistocene stack of 57 globally distributed benthic δ18O records. *Paleoceanogr.* **20**, PA1003, 10.1029/2004PA001071 (2005).

Lloyd, J. M. Modern Distribution of Benthic Foraminifera From Disko Bugt, West Greenland. *J. Foramin. Res.* **36**, 315–331, 10.2113/gsjfr.36.4.315 (2006).

Lloyd, J. M. et al.. A 100 yr record of ocean temperature control on the stability of Jakobshavn Isbrae, West Greenland. *Geology* **39** (9), 867-870. http://dx.doi.org/10.1130/G32076.1 (2011).

Martinson, D. G. *et al.* Age dating and the orbital theory of the ice ages: development of a high-resolution 0 to 300,000-year chronostratigraphy, *Quatern. Res.* **27**, 1-29 (1987).

Möller, P., Fedorov, G., Pavlov, M., Seidenkrantz, M.-S. & Sparrenbom, C. Glacial and palaeoenvironmental history of the Cape Chelyuskin area, Arctic Russia. *Polar Res.* **27**, 222–248 (2008).

Polyak, L. *et al.* Benthic foraminiferal assemblages from the southern Kara Sea, a river-influenced arctic marine environment, *J. Foramin. Res.* **32**, 252–73 (2002).

Rashid, H., Hesse, R. & D. J. W. Piper, D. J. W. Evidence for an additional Heinrich event between H5 and H6 in the Labrador Sea, *Paleoceanogr.* **18** (4), 1077, 10.1029/2003PA000913 (2003).

Rasmussen, T. L., Thomsen, E., Labeyrie, L. & van Weering, Tj. C. E. Circulation changes in the Faeroe-Shetland Channel correlating with cold events during the last glacial period (58 –10 ka). *Geology* **24** (10), 937–940 (1996).

Rasmussen, T. L., Oppo, D. W., Thomsen, E. & Lehmann, S. J. Deep sea records from the southeast Labrador Sea: ocean circulation changes and ice-rafting events during the last 160,000 years, *Paleoceanography* **18** (1), 1018, 10.1029/2001PA000736 (2003).

Reimer, P.J. & Reimer, R.W. 2001. A Marine Reservoir Correction Database and On-Line Interface. *Radiocarbon* **43** (2A), 461-463. 10.1017/S0033822200038339; supplemental material URL: http://intcal.qub.ac.uk/marine/ (2001).

Reimer, P. J. *et al.* IntCal13 and Marine13 radiocarbon age calibration curves 0–50,000 years cal BP. *Radiocarbon* **55**, 1869–1887 (2013).

Rytter, F., Knudsen, K. L., Seidenkrantz, M.-S. & Eiríksson, J. Modern distribution of benthic foraminifera on the North Icelandic shelf and slope. *J. Foramin. Res.* **32**, 217–244 (2002).

Schnitker, D. Quaternary deep-sea benthic foraminifers and bottom water masses, *Ann. Rev. Earth Planet. Sci.*, 8, 343-370 (1980).

Seidenkrantz, M.-S. *Cassidulina teretis* Tappan and *Cassidulina neoteretis* new species (Foraminifera): stratigraphic markers for deep sea and outer shelf areas. *J. Micropalaeontol.* **14**, 145–157 (1995).

Seidenkrantz, M.-S. Benthic foraminifera as palaeo sea-ice indicators in the subarctic realm – examples from the Labrador Sea – Baffin Bay region. *Quatern. Sci. Rev.* **79**, 135–144, 10.1016/j.quascirev.2013.03.014 (2013).

Sen Gupta, B.K. & Machain-Castillo, M.-L. Benthic foraminifera in oxygen-poor habitats. *Mar. Micropaleontol.* **20** (3-4), 183-201 (1993).

Sheldon, C. *et al.* Ice stream retreat following the LGM and onset of the west Greenland current in Uummannaq Trough, west Greenland. *Quatern. Sci. Rev.* **147**, 27–46, 10.1016/j.quascirev.2016.01.019 (2016).

Simon, Q., Hillaire-Marcel, C., St-Onge, G. & Andrews, J. T. North-eastern Laurentide, western Greenland and southern Innuitianice stream dynamics during the last glacial cycle. *J. Quatern. Sci.* **29** (1), 14-26, 10.1002/jqs.2648 (2014).

Steinsund, P.I., Polyak, L., Hald, M., Mikhailov, V. & Korsun, S. Distribution of calcareous benthic foraminifera in recent sediments of the Barents and Kara Sea. In: Steinsund, P. I. *Benthic Foraminifera in Surface Sediments of the Barents and Kara Seas: Modern and Late Quaternary Application.* Ph.D. thesis, Department of Geology, Institute of Biology and Geology, University of Tromsø, Norway (1994).

Stern, J. V. & Lisiecki, L. E. North Atlantic circulation and reservoir age changes over the past 41,000 years. *Geophys. Res. Lett.* **40**, 3693–3697, 10.1002/grl.50679 (2013).

Stoner, J. S., Channell, J. E. T & C. Hillaire-Marcel, C. The magnetic signature of rapidly deposited detrital layers from the Labrador Sea: relationship to North Atlantic Heinrich layers, *Paleoceanogr.* **11**, 309-325 (1996).

Svensson, A. *et al.* The Greenland Ice Core Chronology 2005, 15-42 ka. Part 2: comparison to other records. *Quatern. Sci. Rev.* **25**, 3258-3267 (2006).

Svensson *et al.* A 60 000 year Greenland stratigraphic ice core chronology. *Clim. Past* **4**, 47-57 (2008).

Thornalley, D. J. R., McCave, I. N. & Elderfield, H. Tephra in deglacial ocean sediments south of Iceland: Stratigraphy, geochemistry and oceanic reservoir ages, *J. Quatern. Sci.* **26** (2), 190–198. 10.1002/jqs.1442 (2011).

Weber, M.E. *et al.* Derivations of δ18O from sediment core log data: implications for millennial-scale climate change in the Labrador Sea, *Paleoceanogr.* **16** (5), 503-514 (2001).
